# Measuring the Electrical Status of the Bionic Ear. Re-thinking the Impedance in Cochlear Implants

**DOI:** 10.3389/fbioe.2020.568690

**Published:** 2020-09-18

**Authors:** Federico A. Di Lella, Matias Parreño, Florencia Fernandez, Carlos M. Boccio, Sebastián A. Ausili

**Affiliations:** ^1^Department of Otolaryngology, Hospital Italiano, Buenos Aires, Argentina; ^2^Department of Otolaryngology, University of Miami, Miami, FL, United States

**Keywords:** cochlear implants, electrical stimulation, electrical impedance, impedance subcomponents, voltage telemetry

## Abstract

As in any biophysical electrode-tissue environment, impedance measurement shows a complex relationship which reflects the electrical characteristics of the medium. In cochlear implants (CIs), which is mostly a stimulation-oriented device, the actual clinical approach only considers one arbitrary time-measure of the impedance. However, to determine the main electrical properties of the cochlear medium, the overall impedance and its subcomponents (i.e., access resistance and polarization impedance) should be described. We here characterized, validated and discussed a novel method to calculate impedance subcomponents based on CI measurement capabilities. With an electronic circuit of the cochlear electrode-tissue interface and its computational simulation, the access resistance and polarization impedance were modeled. Values of each electrical component were estimated through a custom-made pulse delivery routine and the acquisition of multiple data points. Using CI hardware, results fell within the electronic components nominal errors (± 10%). Considering the method’s accuracy and reliability, it is readily available to be applied in research-clinical use. In the man-machine nature of the CI, this represents the basis to optimize the communication between a CI electrode and the spiral ganglion cells.

## Introduction

A cochlear implant (CI), also known as “the bionic ear,” is a medical electronic prosthesis that can be precisely controlled to stimulate the auditory nerve and restore the hearing sense. The evaluation of CI functioning is facilitated by various analysis tools, one of the most important is the electric impedance measurement. While it is impossible to directly assess impedance, its values can be obtained by measuring voltage, as provided by Ohm’s law. In CIs, this measurement is performed by using a protocol known as “voltage telemetry” (Hughes, 2013; Wolfe, 2017).

To obtain the electric potential difference at a certain point in time, the CI sends a constant current iso-biphasic pulse and retrieves the measured voltage (French, 1999; von Rohr, 2011). This metric provides important clinical information about the device and individual electrode function, in both intra and post-operative patient’s appointments. In today’s CI standard of care, it is mainly used to investigate the electrode’s overall function (Paasche et al., 2006), detect problems such as short-circuit or open circuit (Goehring et al., 2013), guide audio-processors fitting (Khater et al., 2015), and determine power consumption (Newbold et al., 2015). For example, intra-operatively this measure checks the integrity of the device after surgical manipulation. Post-operatively is also performed at the beginning of every CI-fitting appointment, stablishing the compliance range for the electrical stimulation of the auditory nerve.

The interface between the CI electrode and the spiral ganglion cells is critical for the transmission of information via electro-neural stimulation and consequently, a crucial research area in which improvements can be made (Saunders et al., 2002). According to several authors, the impedance reflects the electrical status of the complex electrode-tissue relationship (Ni et al., 1992; Hughes et al., 2001; Tykocinski et al., 2005). However, the actual clinical impedance measurement provides very limited information to that end, as it was developed for a different purpose. The clinical approach is based on a single voltage measurement and the predefined settings (i.e., voltage evaluation) significantly differ along CI manufacturers (Hughes, 2013; Wolfe, 2017). This makes the actual approach not specifically useful to explore the characteristics of the electro-tissue interface. A complete understanding of the impedance and its subcomponents could provide insights of the actual endocochlear nature around the electrode, extending its use beyond the actual clinical implementation.

Voltage response measurement and impedance subcomponents calculations were reported *in vitro* (Newbold et al., 2004, 2010; Giardina et al., 2018), in animal models (Smith and Finley, 1997; Tykocinski et al., 2001; Newbold et al., 2014) and in humans (Tykocinski et al., 2005; Newbold et al., 2014; Di Lella et al., 2019). Smith and Finley (1997) described the influence of the electrode configuration and electrical stimulation in the complex interface between electrode and neural target in cats. Based on the same animal model, Tykocinski et al. (2001) described the two components of the total impedance, the access resistance and the polarization impedance. Later, Newbold et al. (2014) reported a stimulus-induced reduction in impedance. More recently, Giardina et al. (2018) measured impedance subcomponents *in vitro* using Advanced Bionics, Ltd. hardware. Furthermore, Di Lella et al. (2019) described impedance subcomponent calculation *in vivo* based on voltage telemetry using Cochlear, Ltd. CIs. However, despite the existing literature, methodological details, specific setup configurations and measurement validation are lacking, which restrain these measurements in the clinical setting.

Our study is an extension of the work done by Di Lella et al. (2019) where the impedance subcomponents in cochlear implant users were measured. This study describes the method in detail to recreate the complex morphology of the voltage response for Cochlear, Ltd. devices. This is based completely – and solely – on the CI hardware, without requiring external elements. Unlike actual clinical impedance measures, we extract the impedance subcomponents for a better description of the electrode-tissue relationship. Moreover, we evaluate the accuracy of the subcomponent assessment of the method employing computational circuit simulation and *in silico* electronic hardware. By providing this characterization, our method is not only better supported but, more importantly, translationally ready to be applied in real CI users.

## Materials and Methods

### Modeling the Electrode-Tissue Interface

**FIGURE 1:**
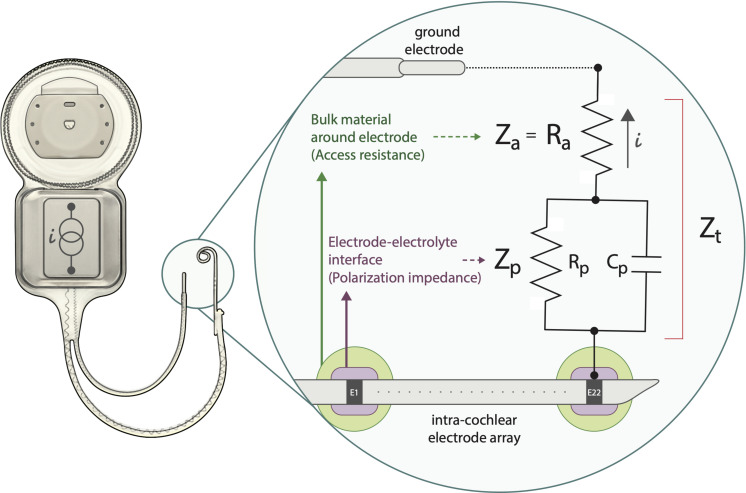
Schematic representation model for the electrode-tissue interface. Z_t_: total impedance between intra-cochlear and ground electrodes. R_a_: access resistance, Z_p_: polarization impedance which includes R_p_: polarization resistance and C_p_: polarization capacitance. i: current flow generated by a constant current source. Note that Z_t_ = Z_a_ + Z_p_. E1-E22: illustrate intracochlear electrodes.

The electrical medium characteristics of the electrode-tissue interface in the inner ear can be modeled with an equivalent electrical circuit (Vanpoucke F. et al., 2004; Vanpoucke F.J. et al., 2004; Tykocinski et al., 2005; Newbold et al., 2010; Mesnildrey et al., 2019). The standard model of a biopotential electrode used for the transduction of ionic current into electric current (both for stimulation and recording) closely recreates the electrical behavior of the electrode-tissue interface in the cochlea. This model facilitates its understanding and makes the model’s equations accessible. In this study, we adopted an existing model where the overall impedance Z_t_ includes an access impedance Z_a_ and a polarization impedance Z_p_, being Z_t_ = Z_a_ + Z_p_ (Figure 1) (Vanpoucke F. et al., 2004; Tykocinski et al., 2005; Newbold et al., 2010). Briefly, an access resistance (R_a_) is in series with a parallel capacitor (C_p_) and a resistor (R_p_). Physiologically, R_a_ represents the bulk surrounding tissue around the electrode inside the cochlea, including fibrous tissue and new bone growth. The sub-component Z_p_ (R_p_ and C_p_) arises from the narrow layer on the surface of the electrode, the electrode-electrolyte interface. C_p_ models the behavior at the electrolyte interface, while the faradaic resistance R_p_ is associated with the transition from electrical to ionic charge carriers. As a whole, Z_p_ is considered a consequence of electrochemical effects, including deposits of electrically charged proteins that modify its distribution with electrical stimulation (Tykocinski et al., 2005).

### Setup Configuration

**FIGURE 2:**
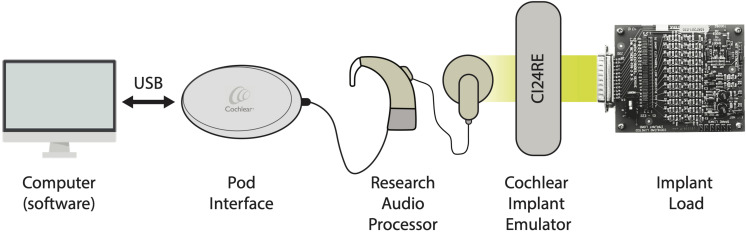
Illustration of the setup configuration chain.

An illustration of the overall setup chain is depicted in Figure 2. In detail, a custom-made software was designed specifically to perform the measurements and obtain the data. Delphi^®^ (Embarcadero, Inc., Austin, TX, United States) programming language together with the dynamic link libraries (DLLs) provided by Cochlear, Ltd. were implemented to communicate with the Nucleus Interphase Communicator (NIC). This software was compiled to run under the Microsoft Windows operating system (Windows 7^®^ and later).

A Cochlear Freedom Speech processor (research firmware ver. 0102E00F02) was connected to the Programming Pod Interface, providing the input to the CI (CI24RE) emulator. The implant load (IL) was coupled to the electrode’s terminals of the implant emulator (via a 25-way D connector) and served as a cochlea simulator to measure and compute the impedances. Each *in silico* electrode routing in the IL is defined by the circuit shown in Figure 1 (a R_a_ in series with a parallel C_p_ and R_p_).

For the purpose of validation, two different ILs were implemented. For the clinical approach validation, we measured the IL provided by Cochlear, Ltd. In this circuit R_a_ varies from 3 to 10 kΩ along electrodes 1 to 22, respectively, and Z_p_ remains constant (C_p_ = 100 nF and R_p_ = 1 MΩ). For the validation of the subcomponents R_a_ and C_p_ we employed a custom IL hardware. Its design allows to fix R_a_ while varying C_p_ (R_a_ validation) and vice versa (C_p_ validation). The subcomponent R_a_ varied from 3 to 9.3 kΩ, C_p_ ranged between 2.8 and 54 nF and R_p_ = 1 MΩ. All electronic components have a tolerance of ± 10%. In order to work in a relevant range, the selected nominal values included the reported of *in vivo* studies (Tykocinski et al., 2005; Di Lella et al., 2019; Mesnildrey et al., 2019). In all cases, the presented voltage value is an average of four consecutive measures. Due to the negligible variation in the measures (±0.001 V), figures only depict the mean voltage value.

We also replicated the custom ILs with virtual computational circuits in MATLAB Simulink (MathWorks, Natick, MA, United States). The input pulses were driven by a current source and the same subcomponents were modeled. The complete waveform was retrieved from the virtual simulation and verified with our fitting on real measurements.

### Clinical Impedance Measurement

**FIGURE 3:**
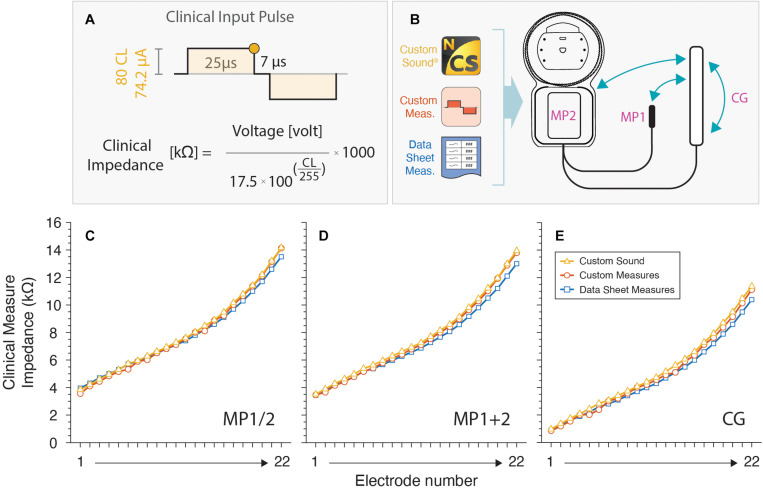
Clinical impedances measurements. **(A)** Input pulse configuration used. The round colored dot at the trailing edge of the first positive pulse is the recording voltage time used for the clinical impedance calculation. **(B)** Custom sound and our custom-made software are used for different modes. **(C–E)** Values are plotted for all electrodes and compared against a datasheet.

To validate the accuracy of our measure, we replicated the clinical voltage telemetry. For that purpose, Cochlear, Ltd. clinical software and IL data sheet were contrasted to our measures. Overall impedances (Z_t_) were obtained with Cochlear Custom Sound Suite^TM^ (version 5.2). In this software the input biphasic pulse is predefined by the manufacturer with 80 current level (CL) (or 74.2 μA), 25 μs of pulse width (PW) and interphase gap (IPG) of 7 μs (Figure 3A; Hughes, 2013; Wolfe, 2017; Cochlear Limited, 2019). The recording time used for this clinical Z_t_ is also predefined to be at the trailing edge of the positive pulse-phase. The equation in Figure 3A shows the Ohm’s law equation for the clinical impedance calculation. Here, the numerator is the measured voltage (in volt) at 25 μs and the denominator the analytical conversion from CL to microamperes (according to Cochlear, Ltd.). Note that the fraction is multiplied by 1000 to represent the result in kΩ. To strengthen the validation measure, we also compare impedances varying stimulation modes (Figure 3B): monopolar MP1, monopolar MP2, monopolar MP1+2, and common ground (CG).

### Stimulation Pulses Parameters and Voltage Response Wave

**FIGURE 4:**
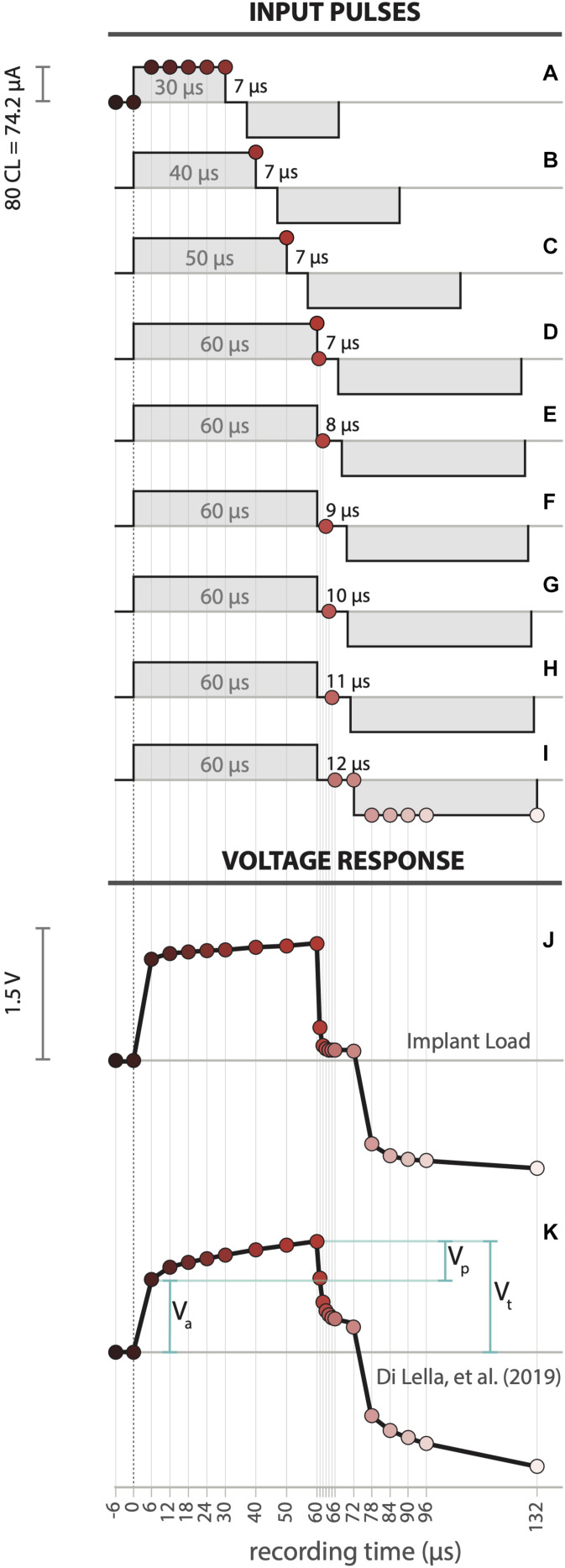
**(A–I)** Detailed pulse sequence design to obtain all voltage samples. Voltage response recorded waveforms for electrode 4 in **(J)** Implant Load and **(K)** an example CI user (Di Lella et al., 2019). Where V_a_: access voltage, V_p_: polarization voltage, V_t_: overall electrode voltage.

The latest Cochlear, Ltd. CI chipset (CIC4) allows up to 14 voltage measurements in different time points for a given pulse. Some of those time points are fixed by hardware, but others depend on the PW and IPG. Therefore, to acquire more points and reconstruct the voltage morphology, several biphasic pulses were used (Figure 4). To obtain the negative-lead voltages, a polarity change was applied. The positive to negative transition is determined at the beginning of the negative phase of stimulus. Note that, although the complete waveform was reconstructed, only voltages from the positive pulse-phase were used to calculate impedance subcomponents.

Under electrical hearing, pulse parameters limits are governed by sound perception thresholds and hearing discomfort. This depends on the pulse’s overall energy and is directly related to both the PW and CL. However, voltage telemetry can be achieved without sound perception, making this measurement convenient and simple for the CI user. Low current pulses with 100 CL and 25 μs are inaudible for most but not all CI recipients (Wolfe and Schafer, 2014). Based on our preliminary experience, CL below 100 units and longer PW around 50 μs do not produce sound perception. Moreover, the built-in analog to digital (AD) amplifier of the CI has limited resolution (0 to 10 volt @16 bit) defining minimum parameters for proper sensitive measures. The pulse current level was set at 80 (74.21 μA) and PW and IPG were modified sequentially to accommodate between sub-threshold sound perception and voltage wave resolution. Points 1 to 7 were collected using pulses with 30 μs PW and 7 μs IPG (Figure 4A). Points 8 to 11 were measured using pulses with increasing PW length, from 40 to 60 μs in steps of 10 μs with a fixed 7 μs IPG (Figures 4B–D). For the subsequent measures the PW was fixed to 60 μs and varied the IPG. For points 12 to 16, we recorded with IPG that increases from 7 to 12 μs in 1 μs steps (Figures 4E–I). Last, points 17 to 22 were determined by using 12 μs IPG (Figure 4I).

A total of 22 voltage points (Figures 4J,K) are extracted from the pulse sequence previously described. This experimental design allows the recreation of the voltage waveform covering −6 to 132 μs. The overall morphology shows a clear consistency with the proposed model (Tykocinski et al., 2005). The abrupt rise in the voltage at the onset of the current pulse corresponds to the resistive component of the circuit (access voltage, V_a_). This is followed by a slowly rising voltage limb, which represents the capacitive component (polarization voltage, V_p_). The overall electrode voltage is the sum of these values (V_t_ = V_a_+V_p_).

### Calculation of Access Resistance and Polarization Impedance

Impedance can be studied using a phasor transform or in the time domain. The phasor transform is represented by a real (resistive) and an imaginary (reactive) components and is not a function of time. By the study of the time variant voltage waveform morphology of a resistor-capacitor circuit, it is possible to approximate the magnitudes of its subcomponents. We here used the time domain approach, also described in Tykocinski et al. (2005) and Giardina et al. (2018).

From the adopted electrical model, the relation between the overall impedance (Z_t_), access resistance (R_a_) and polarization impedance (Z_p_) are well-known and can be mathematically described as follow:

(1) $$Z_{t}\,\left(t\right)=Z_{a}+Z_{p}\,\left(t\right)$$

with

(2) $$Z_{a}=\frac{V_{a}}{i}=R_{a}$$

(3) $$Z_{p}\,\left(t\right)=\frac{V_{p}\,\left(t\right)}{i}=R_{p}\cdot \left(1-e^{\frac{-t}{R_{p}.C_{p}}}\right)$$

The access resistance (R_a_) is simply the quotient of the measured access voltage (V_a_; Figure 4K) with the current pulse amplitude (Eq. 2). Unlike R_a_, Z_p_ varies over time. Therefore, the total impedance can be determined as:

(4) $$Z_{t}\,\left(t\right)=R_{a}+R_{p}\cdot \left(1-e^{\frac{-t}{R_{p}.C_{p}}}\right)$$

We fitted Eq. 4 to our data points with R_p_ and C_p_ as free parameters. This was achieved by minimizing the sum-squared deviation using iterative least squares estimation in MATLAB (MathWorks, Natick, MA, United States). The effect of the access resistance (R_a_) on the voltage waveform is instantaneous once the pulse is delivered. However, due to the hardware limitations we can only record with a 6 μs offset. Therefore, we estimated R_a_ at 0 μs by an extrapolation of the fitting.

## Results

### Clinical Impedance

To validate our measurement tool, we compared the results of the Custom Sound with our custom-made software. Results were also verified with provided values by Cochlear, Ltd. IL data sheet. Results are depicted for MP1 and MP2 (Figure 3C), MP1+2 (Figure 3D), and CG (Figure 3E) coupling modes.

Impedance curves are practically overlapped showing negligible errors along IL electrodes. This comparison serves as a strong validation for the design of our custom measurement tool.

### Complete Voltage Response Wave

Full voltage response wave was obtained for each electrode of the IL in MP1 coupling mode (see Figure 3B). The IL voltage waveform (Figure 4J) showed a significant similarity with a real CI user measurement (Figure 4K) Di Lella et al. (2019). Moreover, both curves relate to the pattern of the modeled electrical circuit (see Figure 1). The described pulse transmission and voltage telemetry acquisitions were completed in approximately 1 min for all electrodes.

### Impedance Subcomponents

#### Theoretical Analysis

**FIGURE 5:**
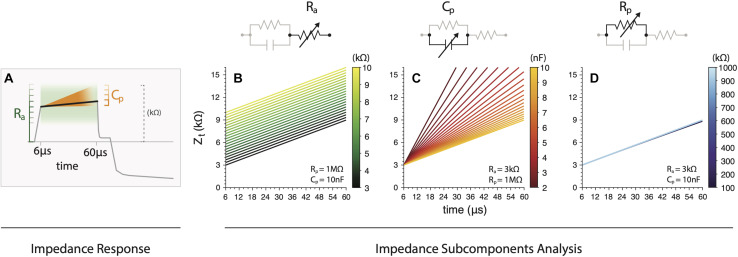
**(A)** Illustration of the main effects of subcomponents variations (R_a_ and C_p_) on the overall impedance. **(B)** R_a_ contribution to the abrupt rise of Z_t_ on its onset. **(C)** Variations of Cp strongly affects the slope of the slowly rising polarization component limb with an inverse relation. **(D)** Variations on R_p_ showing negligible modification on Z_t_.

For a better understanding of the subcomponents and its relationship with Z_t_, we varied R_a_, C_*p*_ and R_p_ in Eq. 4 and analyzed its results. This was modeled for 6 to 60 μs range, which is the main region for subcomponents calculation. The main two components that play a major role modifying the Z_t_ curve are R_a_ and C_p_ as it’s illustrated in Figure 5A. First, we varied R_a_ between 3 and 10 kΩ while maintaining R_p_ = 1 MΩ and C_p_ = 10 nF (Figure 5B). R_a_ linearly modifies the abrupt rise of Z_t_ at its onset. As R_a_ increases higher Z_t_ offsets are seen. Secondly, we ranged 2 nF ≤ C_p_ ≥ 10 nF with R_a_ = 3 kΩ and R_p_ = 1 MΩ (Figure 5C). We observed that Cp strongly affects the slope of the slowly rising polarization component limb with an inverse relation. Finally, we used R_a_ = 3 kΩ and C_p_ = 10 nF while varying 100 kΩ ≤ R_p_ ≥ 1000 kΩ (Figure 5D). Despite the large R_p_ variation there was negligible modification on Z_t_, showing no overall impact. Therefore, we excluded R_p_ from the subcomponent analysis due to its small variation and very poor informative use. Note, however, that this does not affect the estimation of the other circuit elements (Mesnildrey et al., 2019). Moreover, *in vitro* CI measurements also yielded an extremely high estimation for R_p_ (>10^15^ Ω; Mesnildrey et al., 2019) suggesting that no current passes through this resistor. This effect is intrinsically related to the metal-electrolyte interface, meaning that the kinetics of the dissolution of the platinum electrode into the electrolyte is extremely slow (Wieckowski, 1999).

#### Experimental Analysis

**FIGURE 6:**
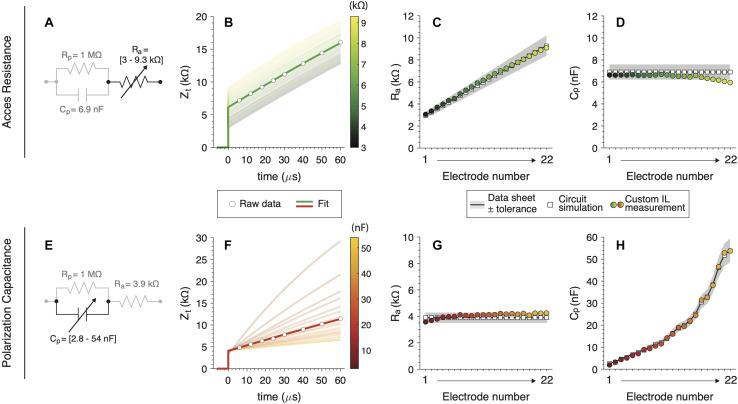
Subcomponents validations measurements for **(A)** a variable R_a_ circuit and **(E)** a variable C_p_ circuit. **(B,F)** Raw measures and fitting for one IL electrode example (dark line). Shadowed lines illustrate the rest of the electrode’s fits. **(C,G)** R_a_ and **(D,H)** C_p_ calculation for custom IL hardware (colored circles), (white squares) and hardware nominal values with their tolerance (black line with gray patch).

Based on this previous theoretical analysis, R_a_ and C_p_ were inferred for all electrodes from the custom IL. Our method outcomes were contrasted with the corresponding electrical hardware and virtual circuit simulation values (Figure 6). To measure isolated values of R_a_ and C_p_ ensuring its correct validation, we first fixed C_p_ and R_p_ while varying only R_a_ (Figure 6A) and then fixed R_a_ and R_p_ varying C_p_ (Figure 6E). Examples of raw measured data and their corresponding Z_t_ fitting are depicted in Figures 6B,F.

Overall, our analysis showed high accuracy for R_a_ as well as C_p_ subcomponents. All measures have small errors and fall within the electrical component’s tolerance range in most cases. We did observe small drops in C_p_ values for higher electrodes (Figure 6D) and in R_a_ for the first electrodes (Figure 6G) when those subcomponents are fixed. This effect was only observed for the combination of R_a_ and C_p_ that yields high Z_t_ (higher clinical values than usual (Hughes, 2013). These drops were not observed when fitting the theoretical simulated circuits.

## Discussion

### A Novel Method

To the author’s knowledge, this is the first report with a complete description, analysis and validation of the electrical CI impedance’s subcomponents measurement for standard Cochlear, Ltd. devices. This protocol ensures that all parameters are measured only using the CI, making it readily available for clinical research purposes. We also ensured that all measures include the known impedance values measured in real CI users. As impedance subcomponents are related with the electrode-tissue interface, they can be exploited to improve CI stimulation. In the man-machine nature of the CI, this represents the basis to optimize the communication between a CI electrode and the spiral ganglion cells.

In electrical circuits, the impedance is normally assessed with the support of an external access tool, where a continuous voltage recording provides high measurement resolution. Since direct intracochlear electrodes measurement is not plausible *in vivo*, we elaborated a novel technique only based on the CI hardware capabilities. We demonstrated that a high resolution and accuracy can be achieved via the CI telemetry communication protocol. In other words, we ‘reversed-engineer’ the *in silico* black-box, which gives us the opportunity to similarly ‘unblind’ the electrical characteristics of the electrode-tissue interface within the implanted inner ear.

The adopted electrical electrode-tissue interface model (Tykocinski et al., 2005) showed high correlation between its theoretical electrical behavior and our test-bench results. We also highlight the most important subcomponents to be considered for future analysis (i.e., R_a_ and C_p_), due to the negligible variation of Z_t_ over a wide range of R_p_ (Figure 5C). Thus, we observe that the electrical electrode-tissue interface is mostly driven by R_a_ and C_p_, making these subcomponents the most relevant variables. Other modified models have been proposed to describe the electrical behavior of this interface (e.g., Duan et al., 2004; Franks et al., 2005; Mesnildrey et al., 2019). Usually, accurate biophysical predictions involve complex representations with a high number of elements in the modeling framework. However, note that even with the simplified model adopted in this study, we observed similar voltage waveforms between *in vivo* (Di Lella et al., 2019) and *in silico* (see Figures 4J,K). Furthermore, since this simple model is mathematically very well-described, calculations of each circuit subcomponent can be quickly achieved.

Moreover, our custom design software successfully measures and processes impedance subcomponents. The novel approach here described is ready to be implemented in CI users as it is (as also demonstrated in Di Lella et al., 2019), making this approach readily useful for future applied research in CI users. This study also serves as a validation document given the presented evidence and proven correlation between objective measures, real electronic components in IL models, and virtual circuit simulations.

### Toward a Real Clinical Use

The current clinical CI-software measure impedances with a predefined (and almost arbitrary selected) series of parameters. Only one biphasic pulse is used as input (e.g., see Figure 3A for Cochlear, Ltd.) and a single voltage data point is measured from the complex voltage electrode-tissue response. Through ohm’s law, the retrieved voltage (V_t_) is converted into impedance (Z_t_) and shown on the clinical software. However, the variable Z_t_ is determined by the main following variables:

• *Measurement Time.* As determined in Eq. 4, the capacitive component of the polarization impedance (Z_p_) generates an asymptotic growing curve. Therefore, Z_t_ systematically changes from Z_a_ to Z_a_ + Z_p_.
• *Coupling Mode.* The configuration of where the circuit’s ground is set modulates the overall measure of Z_t_. This was also observed in our custom software validation (Figures 3C–E).
• *Electrode design.* The electrode surface dimensions (i.e., area of the physical platinum electrode) also impacts and contributes to Z_t_. For example, with smaller electrode surfaces, higher Z_t_ values are expected (Hughes, 2013).
• *Input biphasic pulse.* As Z_t_ increases over time, the shorter the input PW, the smaller the Z_t_ captured (and vice versa).

Impedance subcomponent calculations require precise measurement capabilities. This is directly affected by the following CI-related issues:

• *CI hardware-related issues*. In all measurement oriented devices the internal circuitry defines the intrinsic error and uncertainty of its measure (Horowitz and Hill, 2015). This is of importance in CI devices, which are not specifically designed to perform very precise measures. In our results, C_p_ (electrodes > 17, Figure 6D) and R_a_ (electrodes < 4, Figure 6G) showed a measurement offset which we attributed to CI hardware limitations. This effect was not observed in our virtual circuit simulation fitting. More research should be done to describe the range of Z_t_ to compute impedance subcomponents through the measurement capabilities of the CI.
• *CI software-related issues*. The CI software platform controls and defines the voltage measurement protocol. For example, Cochlear, Ltd. programming library tool only retrieves one voltage measure per pulse. This forces to employ a pulse sequence routine (see Figures 4A–I) which modulates the electrode-electrolyte characteristics, Z_p_ (Newbold et al., 2014). Moreover, the implemented software tools do not allow to perform voltage measures from pulse onset to 6 μs. This clearly introduces a measurement offset in R_a_.

As here discussed, this measure involves device-related variables that are not related to the patient’s specific clinical status. Therefore, the so-called “clinical” impedance is far from being a representative clinical measure. The only useful interpretation of this value is when compared within the subject’s measurement (e.g., over time), only if no internal change of components was done.

CI technology brings the unique possibility to assess the relation between the electrode and the endo-cochlear medium by providing intra-cochlear measurements. Obviously, the medium properties are independent of the utilized device. Our analysis focuses on the impedance subcomponents (R_a_ and C_p_), which, unlike Z_t_, are independent of device-related issues. In other words, by adopting the proposed procedure it is possible to associate the impedance to an effective clinical and useful measure.

The adoption of impedance subcomponents is a promising field to better assess the implanted cochlear health. At present, one of its clinical implementations was oriented to report changes in the cochlear medium after implantation (Tykocinski et al., 2005; Di Lella et al., 2019). Future electrode’s design can be based on the electrode-tissue relationship and the stimulation protocol might be optimized according to certain endo-cochlear properties. Moreover, this approach can precisely monitor the impact of drug-releasing electrodes as well as surgical approaches for its insertion. Longitudinal studies with this tool will not only shed some light to a better understanding of the inflammatory response in the implanted inner ear, but also the development of new approaches to enhance CI-hearing performance. Only increasing the knowledge about the living electrical medium between the electrode and the neurons in the cochlea, CI outcomes can be improved.

## Conclusion

This is the first report with a complete and detailed description, analysis and validation of the electrical impedance subcomponents measurement for Cochlear, Ltd. CIs. This was assessed solely through the CI capabilities, which makes it directly available for clinical research purposes. Even though the present method is based on a simplified model of the electrode-tissue electrical interface, *in silico* values were obtained with high accuracy. In conclusion, based on a better description of this human-machine interface, this approach may enhance CI-hearing performance in our implanted patients.

## Data Availability Statement

The raw data supporting the conclusions of this article will be made available by the authors, without undue reservation.

## Author Contributions

FD and SA designed the methodological approach, collected the data, performed the data analysis, and wrote the manuscript. MP and FF supported the data collection and provided critical revision of the manuscript. CB supervised the findings and revised final manuscript. All authors contributed to the article and approved the submitted version.

## Conflict of Interest

The authors declare that this study received equipment from Cochlear, Ltd. They were not involved in the study design, collection, analysis, interpretation of data, the writing of this article or the decision to submit it for publication.

## References

[B1] Cochlear Limited (2019). *Custom Sound EP software Version 5.2 User Guide D1418673 ISS4*, 86.

[B2] Di Lella F. A. De Marco D. Fernández F. Parreño M. Boccio C. M. (2019). In vivo real-time remote cochlear implant capacitive impedance measurements: a glimpse into the implanted inner ear. *Otol. Neurotol.* 40(Suppl. 1), S18–S22. 31225818 10.1097/MAO.0000000000002214

[B3] Duan Y. Y. Clark M. M. Cowan R. S. C. (2004). A study of intra-cochlear electrodes and tissue interface by electrochemical impedance methods in vivo. *Biomaterials* 25 3813–3828. 10.1016/j.biomaterials.2003.09.107 15020157

[B4] Franks W. Schenker I. Schmutz P. Hierlemann A. (2005). Impedance characterization and modeling of electrodes for biomedical applications. *IEEE Trans. Biomed. Eng.* 52 1295–1302. 10.1109/tbme.2005.847523 16041993

[B5] French M. L. (1999). Electrical impedance measurements with the CI24M cochlear implant for a child with *Mondini dysplasia*. *Br. J. Audiol.* 33 61–66. 10.3109/03005364000000100 10219723

[B6] Giardina C. K. Krause E. S. Koka K. Fitzpatrick D. C. (2018). Impedance measures during in vitro cochlear implantation predict array positioning. *IEEE Trans. Biomed. Eng.* 65 327–335. 10.1109/tbme.2017.2764881 29346102 PMC5929978

[B7] Goehring J. L. Hughes M. L. Baudhuin J. L. Lusk R. P. (2013). How well do cochlear implant intraoperative impedance measures predict postoperative electrode function? *Otol. Neurotol.* 34 239–244. 10.1097/mao.0b013e31827c9d71 23295726 PMC3548045

[B8] Horowitz P. Hill W. (2015). *The Art of Electronics.* Cambridge: Cambridge University Press.

[B9] Hughes M. L. (2013). *Objective Measures in Cochlear Implants.* San Diego, CA: Plural Publishing Inc.

[B10] Hughes M. L. Vander Werff K. R. Brown C. J. Abbas P. J. Kelsay D. M. Teagle H. F. (2001). A longitudinal study of electrode impedance, the electrically evoked compound action potential, and behavioral measures in nucleus 24 cochlear implant users. *Ear Hear.* 22 471–486. 10.1097/00003446-200112000-00004 11770670

[B11] Khater A. M. Moustafa M. F. Said A. Fahmy H. S. (2015). An evidence-based guide for intraoperative cochlear implant backup use. *Int. J. Pediatr. Otorhinolaryngol.* 79 1500–1504. 10.1016/j.ijporl.2015.06.037 26231744

[B12] Mesnildrey Q. Macherey O. Herzog P. Venail F. (2019). Impedance measures for a better understanding of the electrical stimulation of the inner ear. *J. Neural Eng.* 16:016023. 10.1088/1741-2552/aaecff 30523898

[B13] Newbold C. Rachael R. Rodney M. Christie H. Dusan M. Robert S. (2010). Changes in biphasic electrode impedance with protein adsorption and cell growth. *J. Neural Eng.* 7:056011 10.1088/1741-2560/7/5/056011 PMC3543851 20841637

[B14] Newbold C. Richardson R. Huang C. Q. Milojevic D. Cowan R. Shepherd R. (2004). An in vitro model for investigating impedance changes with cell growth and electrical stimulation: implications for cochlear implants. *J. Neural Eng.* 1 1218–1227. 10.1088/1741-2560/1/4/005 15876642

[B15] Newbold C. Risi F. Hollow R. Yusof Y. Dowell R. (2015). Long-term electrode impedance changes and failure prevalence in cochlear implants. *Int. J. Audiol.* 54 453–460. 10.3109/14992027.2014.1001076 25766491

[B16] Newbold C. Silvana M. Rachael R. Peter S. Millard R. Robert C. (2014). Impedance changes in chronically implanted and stimulated cochlear implant electrodes. *Cochlear Implants Int.* 15 191–199. 10.1179/1754762813y.0000000050 23998484

[B17] Ni D. Shepherd R. K. Seldon H. L. Xu S. A. Clark G. M. Millard R. E. (1992). Cochlear pathology following chronic electrical stimulation of the auditory nerve. I: normal hearing kittens. *Hear. Res.* 62 63–81. 10.1016/0378-5955(92)90203-y 1429252

[B18] Paasche G. Bockel F. Tasche C. Lesinski-Schiedat A. Lenarz T. (2006). Changes of postoperative impedances in cochlear implant patients: the short-term effects of modified electrode surfaces and intracochlear corticosteroids. *Otol. Neurotol.* 27 639–647. 10.1097/01.mao.0000227662.88840.61 16868511

[B19] Saunders E. Lawrence C. Antje A. William S. Michelle K. Mathias S. (2002). Threshold, comfortable level and impedance changes as a function of electrode-modiolar distance. *Ear Hear.* 23 28S–40S. 11883764 10.1097/00003446-200202001-00004

[B20] Smith D. W. Finley C. C. (1997). Effects of electrode configuration on psychophysical strength-duration functions for single biphasic electrical stimuli in cats. *J. Acoust. Soc. Am.* 102 2228–2237. 10.1121/1.419636 9348680

[B21] Tykocinski M. Cohen L. T. Cowan R. S. (2005). Measurement and analysis of access resistance and polarization impedance in cochlear implant recipients. *Otol. Neurotol.* 26 948–956. 10.1097/01.mao.0000185056.99888.f3 16151342

[B22] Tykocinski M. Duan Y. Tabor B. Cowan R. S. (2001). Chronic electrical stimulation of the auditory nerve using high surface area (HiQ) platinum electrodes. *Hear. Res.* 159 53–68. 10.1016/s0378-5955(01)00320-3 11520634

[B23] Vanpoucke F. Zarowski A. Casselman J. Frijns J. Peeters S. (2004). The facial nerve canal: an important cochlear conduction path revealed by Clarion electrical field imaging. *Otol. Neurotol.* 25 282–289. 10.1097/00129492-200405000-00014 15129106

[B24] Vanpoucke F. J. Zarowski A. J. Peeters S. A. (2004). Identification of the impedance model of an implanted cochlear prosthesis from intracochlear potential measurements. *IEEE Trans. Biomed. Eng.* 51 2174–2183. 10.1109/tbme.2004.836518 15605865

[B25] von Rohr R. (2011). *Cochlear Implant Impedance Telemetry Measurements and Model Calculations to Estimate Modiolar Currents.* Zürich: University of Zurich.

[B26] Wieckowski A. (1999). *Interfacial Electrochemistry: Theory: Experiment, and Applications.* Boca Raton, FL: CRC Press.

[B27] Wolfe J. (2017). *Cochlear Implants: Audiologic Management and Considerations for Implantable Hearing Devices.* San Diego, CA: Plural Publishing Incorporated.

[B28] Wolfe J. Schafer E. C. (2014). *Programming Cochlear Implants.* San Diego, CA: Plural Publishing.

